# CT Radiomics and Whole Genome Sequencing in Patients with Pancreatic Ductal Adenocarcinoma: Predictive Radiogenomics Modeling

**DOI:** 10.3390/cancers14246224

**Published:** 2022-12-16

**Authors:** Ricarda Hinzpeter, Roshini Kulanthaivelu, Andres Kohan, Lisa Avery, Nhu-An Pham, Claudia Ortega, Ur Metser, Masoom Haider, Patrick Veit-Haibach

**Affiliations:** 1 Joint Department of Medical Imaging, Princess Margaret Hospital, University Health Network, University of Toronto, Toronto, ON M5G 2C1, Canada; 2 Department of Biostatistics, Princess Margaret Cancer Centre, University Health Network, University of Toronto, Toronto, ON M5G 2C1, Canada; 3 Division of Biostatistics, Dalla Lana School of Public Health, University of Toronto, Toronto, ON M5T 3M7, Canada; 4 Princess Margaret Cancer Centre, University Health Network, Toronto, ON M5G 2C1, Canada

**Keywords:** computed tomography, pancreatic ductal adenocarcinoma, radiogenomics, whole genome sequencing

## Abstract

**Simple Summary:**

Linking imaging-derived radiomics features to underlying tumor biology and pathogenesis is a developing field of increasing interest, given the wide availability of imaging data in contrast to costs, expenses and logistical issues of molecular analyses. While invasive tissue sampling remains the gold standard for histologic characterization, the usage of noninvasive imaging techniques for diagnosis and detection of specific tumor characteristics could represent a potential additive or eventually an alternative, especially in patients with advanced, inoperable disease or when inaccessible to biopsy. Biomarkers are continuously expanding and several actionable targets have already been identified in pancreatic ductal adenocarcinoma (PDAC), in order to guide clinical decision making and help to develop novel treatment strategies. Our study indicates acceptable correlation of CT-derived radiomics features and driver gene mutations in PDAC.

**Abstract:**

We investigate whether computed tomography (CT) derived radiomics may correlate with driver gene mutations in patients with pancreatic ductal adenocarcinoma (PDAC). In this retrospective study, 47 patients (mean age 64 ± 11 years; range: 42–86 years) with PDAC, who were treated surgically and who underwent preoperative CT imaging at our institution were included in the study. Image segmentation and feature extraction was performed semi-automatically with a commonly used open-source software platform. Genomic data from whole genome sequencing (WGS) were collected from our institution’s web-based resource. Two statistical models were then built, in order to evaluate the predictive ability of CT-derived radiomics feature for driver gene mutations in PDAC. 30/47 of all tumor samples harbored 2 or more gene mutations. Overall, 81% of tumor samples demonstrated mutations in KRAS, 68% of samples had alterations in TP53, 26% in SMAD4 and 19% in CDKN2A. Extended statistical analysis revealed acceptable predictive ability for KRAS and TP53 (Youden Index 0.56 and 0.67, respectively) and mild to acceptable predictive signal for SMAD4 and CDKN2A (Youden Index 0.5, respectively). Our study establishes acceptable correlation of radiomics features and driver gene mutations in PDAC, indicating an acceptable prognostication of genomic profiles using CT-derived radiomics. A larger and more homogenous cohort may further enhance the predictive ability.

## 1. Introduction

Pancreatic ductal adenocarcinoma (PDAC) is an aggressive solid tumor and is associated with poor prognosis with a 5-year survival rate of 6–7% [1]. PDAC is considered the fourth most common cause of cancer-associated death in the United States, reflected by estimated 57,600 new cases and resulting in 47,050 deaths in 2020 [2]. Due to low detection rates at initial stages, the majority of patients are diagnosed with locally advanced or metastatic disease and are not candidates for potential curative surgery, resulting in median survival between 6.1 and 11 months [3].

In recent years, advanced genomic sequencing technologies and molecular profiling contributed to further characterization of recurrent genetic alterations in PDAC, yielding important insights in tumor biology and improved understanding of familial predisposition while delivering potentially valuable prognostic tools and targeted therapeutics approaches. Recent genomic studies of PDAC have identified high-frequency alterations in the function of many key oncogenes and tumor suppressor genes, including KRAS, TP53, SMAD4 and CDKN2A [4,5,6]. Moreover, RNA analyses of PDAC resection specimens have demonstrated gene expression signatures with prognostic and biological relevance [7,8].

Ongoing improvements in imaging modalities, techniques and postprocessing tools have intensified the radiological assessment and management of PDAC. Particularly recent developments of machine learning techniques and the huge growth of computational power has driven the field of radiomics [9]. The principles of radiomics include extraction of high-dimensional data from various sources of medical images, followed by an analysis of different classes of radiomic features, aiming to support clinical decision-making to overcome the limitations of solely visual image interpretation [10]. Previous studies have demonstrated the potential to describe underlying tumor biology of various cancer types using radiomics [11,12,13].

Therefore, decrypting pathogenesis and tumor biology in correlation with imaging-derived radiomics would be highly desirable, in order to guide clinical decision making and help to develop novel treatment strategies, including a targeted therapy approach. Thus, the primary aim of our study was to investigate whether CT-derived radiomics features may correlate with driver mutations from whole genome sequencing of PDAC resection specimens.

## 2. Materials and Methods

Based on a database search in cBioPortal, 55 patients with PDAC who underwent Whipple’s procedure or distal pancreatectomy between June 2008 and November 2015 were identified. Patients were excluded due to the lack of preoperative CT imaging (n = 5) and/or missing whole genome sequencing data (n = 3).

Demographic patient data is provided in Table 1. Our study received institutional review board and local ethics committee approval (CAPCR/UHN REB #: 20-6105). All patients provided a written informed consent prior to the study inclusion for evaluation of genome sequencing.

### 2.1. Image Acquisition

All CT examinations were performed on a 64-slice single source CT scanner (SOMATOM Definition, Siemens Healthineers, Erlangen, Germany). Acquisition parameters were as follows: tube voltage, 120 kVp, maximum allowable tube current set at 200 mAs using automated exposure control (CAREDose 4D), gantry rotation time, 500ms, pitch 1, beam collimation 32  ×  0.6 mm. A bolus of 140 mL of iopromide (Ultravist 370; Bayer Schering Pharma AG, Berlin, Germany) was administered at a rate of 3 mL/s through a 20-gauge angiographic catheter inserted into an antecubital vein. Portalvenous phase images were performed using a bolus tracking technique, 75 s delay after the aortic attenuation at the level of the diaphragm had reached 100 Hounsfield units (HU). Additional arterial phase was performed in 19/47 patients. Images with a transverse pixel size of 1.00 and slice thickness of 5 mm were reconstructed in the axial plane using a soft tissue kernel.

### 2.2. Image Segmentation, Radiomic Feature Extraction and Genomic Data

**Figure 1:**
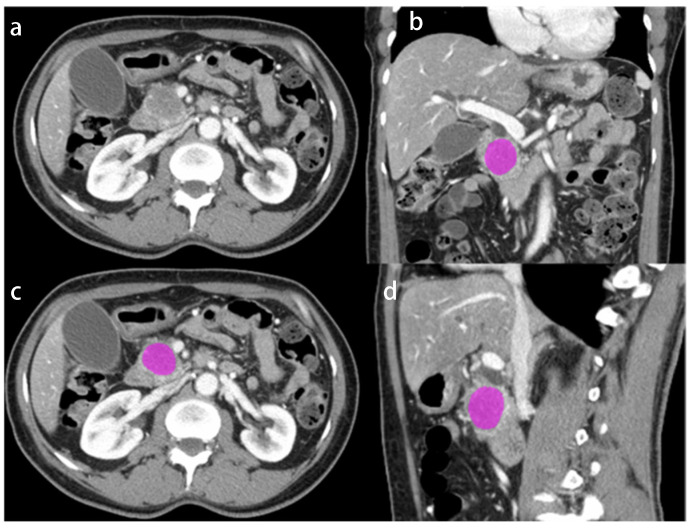
Axial CT image (**a**) and triplanar CT images with segmentation (displayed in pink; **b**, **c**, **d**) in a representative 62-year-old woman with PDAC in the pancreatic head.

Image segmentation and radiomics features extraction was performed with a commonly used open-source software platform (LIFEx, Version 6.30; [14]) by one independent reader with 7 years of experience in oncologic radiology. The contours of the primary pancreatic tumor were manually delineated and segmented in venous phase of the preoperative CT in slice-by-slice fashion (Figure 1).

Next generation sequencing for whole genome sequencing was performed using Illumina HiSeq 2000/2500 reagents and instruments on paired-end library at the Ontario Cancer Research Institute (Toronto, ON, Canada). Data analysis protocol was based on previously described methods [15]. Illumina’s CASAVA software (version 1.8.2) converted the sequencing base calls to fastq format reads. Reads were aligned to the human reference genome (hg19_random) using Burrows-Wheeler Aligner (version 0.6.2) [16]. Somatic single nucleotide mutations were called using both Strelka (version 1.0.7) [17], and MuTect (version 1.1.4) [18], while indels were called using only Strelka (version 1.0.7). ANNOVAR [19] was used to annotate all the final mutation calls. Somatic copy number variation was assessed using CELLULOID [20].

Multiple genes, that have been previously reported as altered in PDCA were collected from a web-based resource (cBioPortal.ca for Cancer Genomics [21,22]) and were categorized as follows: significantly mutated genes, oncogenes, DNA damage repair genes and chromatin modification genes [7,8,23]. The 4 most prevalent genes were then included for statistical analysis (KRAS, TP53, SMAD4, CDKN2A) (Table 2).

### 2.3. Statistical Analysis and Modelling

#### 2.3.1. Feature Selection

To reduce the number of radiomic features fed into the model building, a complete clustering algorithm was used to group correlated features to form a feature dendogram. Prior to clustering, features were normalized using a data-dependent normalization algorithm (R package bestNormalize, Version 1.8.3 [24]).

Features were selected from seven clusters, based on visual inspection of the cluster trees to strike a balance between cluster size and number of clusters. These clusters were independent of mutation status and clustering was performed once for the entire feature set. A reduced set of features was then selected for each mutation by choosing the feature from each cluster with the largest squared Spearman correlation with mutation status. Because our goal was to build a verifiable prediction model independent of our sample, we used raw values for the modelling of normally distributed features and a log transformation for those features with a mean/median ratio of >1.5. This step resulted in a reduced data set of seven features for each mutation.

#### 2.3.2. Model Building

Prior to model building, 1000 cross-validation (CV) sets were selected for internal validation. Each set was comprised of 37 training participants and 10 testing participants, randomly allocated.

From the reduced set of features identified for each mutation all possible subset logistic regression (APS-LR) was performed to determine which combination of variables maximized the Youden Index (Sensitivity + Specificity − 1). APS-LR is computationally intensive, with processing time increasing exponentially with the number of variables under consideration and is possible because of the pre-selection of influential features. Two approaches were used for model building.

Method 1 involved performing APS-LR for each of the 1000 CV training sets and evaluating the model on the corresponding testing set. This approach allowed us to determine the most important features, defined as those appearing most frequently in the list of model predictors.

Method 2 involved performing the APS-LR step once, on the complete dataset to identify the model most predictive of each mutation, fixing the feature set for each mutation. The regression parameters from this model were then re-estimated on each of the 1000 CV training sets and evaluated on the testing sets.

Both methods allow us to inspect the distribution of the statistics associated with the model and therefore to estimate the distribution of the performance statistics, which cannot be achieved with a single testing/training set.

## 3. Results

### 3.1. Baseline Characteristics of the Study Cohort

Overall, 47 patients (21 females, 26 males; mean age 64 ± 11 years, range: 42–86 years) who received preoperative CT imaging and whole genome sequencing of the pancreatic resection specimen were included in our study. No systemic treatment was administered prior to surgery.

At the time of diagnosis, the majority of patients presented with advanced local disease with T3 or T4 PDAC in 42/47 (89%) patients, lymph node metastasis (N1) in 37/47 (78%) patients and M1 disease in 12/47 (26%) patients.

All patients were treated surgically, either with Whipple’s procedure (41/47; 87%) or distal pancreatectomy (6/47; 13%). The majority of patients underwent adjuvant chemotherapy (42/47; 89%) (Table 1).

### 3.2. Whole Genome Sequencing

More than half (30/47) of the analyzed tumor samples harbored 2 or more gene mutations. Overall, 81% of tumor samples demonstrated a missense mutation in KRAS, 68% of samples had alterations in TP53, 26% in SMAD4 and 19% in CDKN2A (Table 2).

### 3.3. Method 1

**Figure 2:**
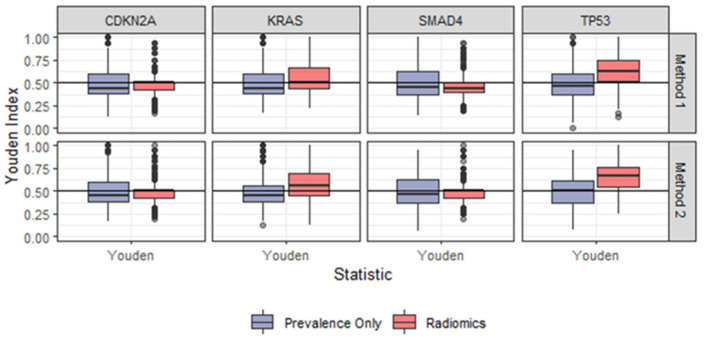
Boxplots demonstrating the Youden Index comparing method 1 (**upper row**) and method 2 (**lower row**).

Method 1 revealed a set of important radiomics features for KRAS, TP53, SMAD4 and CDKN2, respectively (Table 3). Feature importance was calculated as the number of cross-validation models a feature appeared in. The most important features for each gene were as follows: HU_Skewness for both KRAS (number of cross-validation 918/1000) and TP53 (number of cross-validation 981/1000), GLZLM_SZLGE for SMAD 4 (number of cross-validation 743/1000) and NGLDM_Coarseness for CDKN2A (number of cross-validation 584/1000) (Table 3). The Youden Index for model 1 were as follows: 0.50 for KRAS, 0.62 for TP53, 0.44 for SMAD4 and 0.50 for CDKN2A (Table 4 and Figure 2).

### 3.4. Method 2

As compared to the model with varying radiomics features (method 1), the model performance slightly improved with a fixed set of the most important radiomics features (method 2). The most important features for each gene are displayed in Table 5. The Youden Index for method 2 were as follows: 0.56 for KRAS, 0.67 for TP53 and 0.50 for both SMAD4 and CDKN2A (Table 4 and Figure 2).

## 4. Discussion

In our study we assessed the association of CT-derived radiomics features with genetic driver mutations in surgical PDAC resection specimens. The main finding of our study demonstrates an acceptable correlation of the most important radiomics features with TP53 and KRAS gene mutation (Youden index of 0.67 and 0.56, respectively), indicating a reasonable predictive ability of genomic profiles using CT-derived radiomics.

Linking radiomics features to underlying tumor biology and pathogenesis is a developing field of increasing interest, given the wide availability of imaging data in contrast to costs, expenses and logistical issues of molecular analyses. While invasive tissue sampling remains the gold standard for histologic characterization, the usage of noninvasive imaging techniques for diagnosis and detection of specific tumor characteristics could represent a potential additive or eventually an alternative, especially in patients with advanced, inoperable disease or when inaccessible to biopsy. Biomarkers are continuously expanding and several actionable targets have already been identified in PDAC [25,26], partly already guiding clinical decision making and helping to develop novel targeted treatment strategies. Several genomic sequencing studies have also reported poor outcome in the presence of driver gene mutations in the PDAC resection specimen [27,28,29]. Although early stage precancerous lesions already appear to harbor driver genes mutations like KRAS and TP53, suggesting that these gene alterations play an important role in tumor onset and progression, genomic analysis of resection specimens represents only a snapshot of existing mutations [30,31].

Chemotherapy and combined chemo-radiotherapy is used both in adjuvant and neoadjuvant treatment of PDAC, however many patients are either intrinsically resistant or will acquire resistance to these treatments [32]. Therefore, it is crucial to understand related molecular mechanisms and pathogenesis, since specific gene mutations have been identified to contribute to reduced drug sensitivity and effectiveness of alternative therapeutic strategies. For example, a recent study showed that KRAS mutation plays an important role in the metabolic reprogramming of cancer cells, shifting them towards an anabolic metabolism and support unconstrained proliferation, leading to reduced activity of both gemcitabine and paclitaxel [33,34]. Wang et al. [35] also demonstrated resistance to radiotherapy in SMAD4-mutated PDAC through the induction of reactive oxygen species production and increased level of radiation-induced autophagy. This may indicate that the assessment of SMAD4 status represents a vital screening test, not only prior to radiotherapy but also for overall disease estimation, since multiple clinical studies showed that SMAD4 mutated PDAC commonly had distant disease progression as compared to those with intact SMAD4, who more commonly had local dominant disease pattern [36,37]. Several studies demonstrated that functional CDKN2A inactivation caused by mutations and deep deletions were associated with poor prognosis in PDAC patients [38,39,40]. Oshima et al. [41] investigated 106 surgically treated patients with PDAC and discovered that the loss of CDKN2A was significantly associated with lymphatic invasion and postoperative metastases. Another study, however, investigating chemosensitivity profiling of PDAC cell lines and patient-derived organoids, found that CDKN2A inactivation correlated with increased sensitivity to paclitaxel and an active metabolite of irinotecan [38].

In general, radiomics features can be classified into three main categories [42]. Shape features describe semantic and/or geometric properties (e.g., volume, maximum diameter). First order features describe the distribution of individual voxel values without any concern for their spatial relationships (e.g., skewness, kurtosis). Second order features describe the statistical relationship between neighboring voxels, providing a measure of the spatial arrangement of the voxel intensities and hence of intra-lesion heterogeneity (e.g., grey-level co-occurrence matrix, grey-level run length matrix).

Several studies so far investigated the correlation of radiomics feature and gene expression profile in various malignancies with a main focus on Non-Small Cell Lung Cancer (NSCLC) [43,44,45]. For example, Zhang et al. [44] constructed a clinical-radiological-radiomics model (C-R-R), based on the combination of CT radiomics feature signature with clinical and radiological features, aiming to predict epidermal growth factor receptor (EGFR) status among 420 patients with lung adenocarcinoma. The model demonstrated excellent diagnostic performance and high sensitivity in predicting EGFR mutation status. Another study by Digumarthy et al. [46] concluded that CT-derived radiomics features of NSCLC can help distinguish between EGFR positive and wild-type adenocarcinomas.

Further studies focused on the utility of CT radiomics features in PDAC, applied over a wide range of study purposes. Chu et al. [47] determine the utility of CT radiomics features in differentiating PDAC from normal pancreatic tissue in a cohort of 190 patients. The authors concluded that radiomics can be used to differentiate between CT cases from patients with PDAC and healthy control subjects with normal pancreas, using the maximally relevant-features, including gray-level-co-occurrence matrix, summation of the entropy and shape features of the whole pancreas. A similar work by Chen et al. [48] assessed the ability of CT radiomics features to distinguish between patients with and without PDAC in a large retrospective study, including 436 patients. The authors identified several features in the cancerous patch with lower values reflecting image intensity (first order: median, mean and 90th percentile) and higher values for features reflecting heterogeneity (NGTDM busyness, GLDM gray-level nonuniformity and GLDM dependence nonuniformity), consistent with the notion that PDACs typically manifest with heterogenous hypoenhancement on CT. Multiple other studies investigated survival prediction in PDAC using radiomics [49,50,51,52]. For example, Khalvati et al. [51] conducted a multicenter study, including 98 patients, aiming to assess the reproducibility and prognostic value of CT-derived radiomics features for resectable PDAC. The authors identified 2 significant second order features, among those, entropy-related features. Entropy measures the degree of randomness or non-uniformity in the image and it has been hypothesized that it can act as a surrogate for tumor heterogeneity. Notably, the majority of significant radiomics features, which were included in our prediction models consistent of either grey-level zone length matrix (GLZLM) or grey-level run length matrix (GLRLM), indicating spatial voxel distribution with estimation of tumor heterogeneity.

To the best of our knowledge there is only very limited literature investigating the correlation of CT radiomics features and gene expression profile in patients with PDAC. Attiyeh et al. [53] demonstrated a strong correlation of CT derived radiomics features with PDAC genetic alterations. In a cohort of 34 patients, 46% were found to have a mutation in SMAD4 and 83% showed alterations in TP53 expression. The authors found a good discriminatory power between patients with normal and abnormal SMAD4 status using CT-derived radiomics, whereas the radiomics feature model for TP53 mutations did not clearly discriminate between normal and those with altered TP53 status. The results of our study, which included a larger set of patients with PDAC, demonstrated superior predictive ability of TP53 mutation status (Youden Index 0.67). SMAD4 mutation status demonstrated only acceptable predictive signal (Youden Index 0.5), which may be explained by the relative low prevalence of SMAD4 mutations in our cohort (26% vs. 46%). Further, we followed a more holistic approach searching for more than twenty important gene mutations in PDAC, resulting in the inclusion of 4 main driver gene mutations (TP53, KRAS, SMAD4, CDKN2A) in our analysis. This may be of particular importance, since the interaction of synchronous gene mutations plays a crucial role in the development of drug resistances [54].

A further study by Iwatate et al. [55] evaluated in 107 patients with PDAC whether the expression of TP53 could be predicted using machine learning. The results of their study revealed the expression of TP53 to be predictable from CT texture features with a sensitivity of 0.667 and a specificity of 0.813 (AUC 0.795). The results of our analysis demonstrated higher sensitivity for predicting TP53 mutation. Our models revealed a maximal Youden Index of 0.67, with a sensitivity of 0.83 (95%CI 0.50–1.00) and a specificity of 0.50 (95%CI 0.00–1.00). However, the authors only investigated TP53, whereas we pursued a more holistic approach, including 4 main driver gene mutation in PDAC, which adds value to the literature.

As compared to the mentioned prior studies [53,55], we believe that we applied a more extended and more rigorous statistical analysis, reflecting a possible explanation for certain differences. It is common in high-dimensional studies, with many features relative to cases to perform split-validation, where the sample is divided into a training set (typically 80% of cases) and a distinct testing set. This strategy is used to ensure that the built model does not over-fit the data and to provide information about the reproducibility of the findings. Our strategy followed that of Papp et al. [56] whereby the sample was split into 1000 cross validation samples, each containing distinct training and testing sub-samples. We feel this approach is more robust because it enables us to obtain a distribution of the validation statistics and thereby shows the distribution of predictive accuracies, as opposed to a single feature estimate. While a single validation sample may indicate excellent discrimination the most clinically useful results arise when a large proportion of validation samples display high sensitivity and specificity. Additionally, the ability to place some confidence bounds around the reproducibility of the results is gained when multiple validation sets are used.

The following study limitations must be acknowledged. First, there are inherent drawbacks, due to the retrospective nature of the study and the relatively small sample size. Second, we investigated a relative inhomogeneous set of patients consisting of various tumor stages and grades, thus depicting different timepoints in the course of the disease. Third, due the relatively small sample size of our cohort, we did not discriminate between different molecular subtypes of PDAC, although we are aware of clinical and prognostic implications. Fourth, the Youden Index for the included mutations was 0.5–0.67, reflecting acceptable predictive ability, however given applied extended and rigorous statistical analysis, the respective values may demonstrate a reasonable result in this setting.

## 5. Conclusions

In conclusion, our study indicates acceptable correlation of CT-derived radiomics features and driver gene mutations in PDAC. Given small sample size and relative heterogeneity of the study cohort, we believe that a larger and more homogenous cohort may enhance the predictive ability.

## Figures and Tables

**Table 1:** Demographic data of the study cohort.

Characteristics	n = 47
Age (mean ± SD; range)	63.7 ± 10.7 (42–86)
Sex	
Females	21 (45%)
Male	26 (55%)
Current or former smoker	21 (45%)
Race	
Asian	6 (13%)
Non-Asian	41 (87%)
Tumor Grade	
G1	8 (17%)
G2	26 (55%)
G3	12 (26%)
G4	1 (2%)
TNM	
T2	5 (11%)
T3-4	42 (89%)
N1	37 (78%)
M1	8 (17%)
Treatment	
Surgery	47 (100%)
Whipple’s procedure	41 (87%)
Distal Pancreatectomy	6 (13%)
Adjuvant Chemotherapy	42 (89%)

**Table 2:** Prevalence of genomic alterations in PDAC resection specimens. The 4 most prevalent gene mutations are presented in bold.

Genes	Prevalence of Mutations N (%)
Significantly mutated genes	
**KRAS**	38 (81)
**TP53**	32 (68)
**SMAD4**	12 (26)
**CDKN2A**	9 (19)
ARID1A	2 (4)
TGFBR2	1 (2)
NF1	1 (2)
Oncogenes	
BRAF	1 (2)
EGFR	1 (2)
ERBB2	1 (2)
FGFR1	1 (2)
GNAS	1 (2)
MET	1 (2)
PAK4	1 (2)
DNA damage repair genes	
BRCA2	1 (2)
ATM	1 (2)
Tumor suppressor genes	
RNF43	2 (4)
ACVR2A	2 (4)
SMAD3	1 (2)
TSC2	1 (2)
Chromatin modification genes	
KDM6A	1 (2)
KMT2C	3 (6)
PBRM1	1 (2)
SMARCA2	1 (2)
SMARCA4	1 (2)

**Table 3:** Feature importance of method 1 for each gene mutation with a varying set of features. Feature importance was calculated as the number of cross-validation models a feature appeared in 1000 sets.

Feature	KRAS	TP53	SMAD4	CDKN2A
HU_Skewness	918	981		
HU_Q3	860			
GLZLM_LZLGE	690			
GLRLM_LRLGE	664			
GLRLM_SRHGE	578			
GLRLM_LGRE	514			
NGLDM_Contrast	470			
GLZLM_SZHGE		782		
NGLDM_Coarseness		752	423	584
GLCM_Energy		617		
GLZLM_ZLNU		502		
PARAMS_ZSpatialResampling		353		
HU_max		351		
GLZLM_SZLGE			743	397
HU_peakSphere1mL			633	
HUmin			541	465
GLZLM_GLNU			528	561
HU_Q1			491	580
SHAPE_Volume			396	
GLCM_Correlation				526
SHAPE_Sphericity				461

**Table 4:** Statistical parameters (median and 95% CI) for method 1 and method 2 across the cross-validation samples.

	KRAS	TP53	SMAD4	CDKN2A
Model 1				
NPV	0.00 (0.00, 1.00)	0.50 (0.00, 1.00)	0.75 (0.44, 1.00)	0.80 (0.56, 1.00)
PPV	0.86 (0.56, 1.00)	0.75 (0.43, 1.00)	0.00 (0.00, 0.50)	0.00 (0.00, 0.50)
Sensitivity	0.88 (0.57, 1.00)	0.80 (0.44, 1.00)	0.00 (0.00, 0.67)	0.00 (0.00, 0.50)
Specificity	0.00 (0.00, 1.00)	0.50 (0.00, 1.00)	0.86 (0.50, 1.00)	0.89 (0.56, 1.00)
Youden Index	0.50 (0.31, 0.94)	0.62 (0.31, 0.94)	0.44 (0.28, 0.72)	0.50 (0.28, 0.69)
Model 2				
NPV	0.33 (0.00, 1.00)	0.50 (0.00, 1.00)	0.75 (0.50, 1.00)	0.80 (0.56, 1.00)
PPV	0.88 (0.62, 1.00)	0.78 (0.43, 1.00)	0.00 (0.00, 1.00)	0.00 (0.00, 1.00)
Sensitivity	0.88 (0.62, 1.00)	0.83 (0.50, 1.00)	0.00 (0.00, 0.50)	0.00 (0.00, 0.50)
Specificity	0.25 (0.00, 1.00)	0.50 (0.00, 1.00)	0.89 (0.57, 1.00)	0.89 (0.56, 1.00)
Youden Index	0.56 (0.33, 0.97)	0.67 (0.33, 0.94)	0.50 (0.31, 0.69)	0.50 (0.31, 0.75)

**Table 5:** Feature importance of method 2 for each gene mutation with a fixed set of features based on the complete sample.

Feature	KRAS Mutations N (%)	TP53	SMAD4	CDKN2A
HUSkewness	x	x		
HU_Q3	x			
GLZLM_LZLGE	x			
GLRLM_LRLGE	x			
NGLDM_Contrast	x			
GLZLM_SZHGE		x		
NGLDM_Coarseness		x	x	x
GLCM_Energy		x		
GLZLM_ZLNU		x		
GLZLM_SZLGE			x	x
HUpeakSphere1mL			x	x
GLZLM_GLNU			x	x
HU_Q1				x
GLCM_Correlation				x
SHAPE_Sphericity				x

## Data Availability

Not applicable.
